# Underwater and Hyperbaric Medicine as a Branch of Occupational and Environmental Medicine

**DOI:** 10.1186/2052-4374-25-39

**Published:** 2013-12-19

**Authors:** Young Il Lee, Byeong Jin Ye

**Affiliations:** 1 Boryeong public health center, 234, Boryeongnam-ro, Nampo-myeon, Boryeong-si, Chungcheongnam-do 355-882, South Korea; 2 Department of Occupational & Environmental Medicine, College of Medicine, Kosin University, 34-1 Amnam-dong, Seo-gu, Busan 602-702, South Korea

**Keywords:** Underwater and hyperbaric medicine, Occupational and environmental medicine, Barotrauma, Decompression illness, Toxicity of diving gases, Fitness for diving

## Abstract

Exposure to the underwater environment for occupational or recreational purposes is increasing. As estimated, there are around 7 million divers active worldwide and 300,000 more divers in Korea. The underwater and hyperbaric environment presents a number of risks to the diver. Injuries from these hazards include barotrauma, decompression sickness, toxic effects of hyperbaric gases, drowning, hypothermia, and dangerous marine animals. For these reasons, primary care physicians should understand diving related injuries and assessment of fitness to dive. However, most Korean physicians are unfamiliar with underwater and hyperbaric medicine (UHM) in spite of scientific and practical values.

From occupational and environmental medicine (OEM) specialist’s perspective, we believe that UHM should be a branch of OEM because OEM is an area of medicine that deals with injuries caused by physical and biological hazards, clinical toxicology, occupational diseases, and assessment of fitness to work. To extend our knowledge about UHM, this article will review and update on UHM including barotrauma, decompression illness, toxicity of diving gases and fitness for diving.

## Introduction

Due to the recent increase of diving for occupational and recreational purposes, the population of divers is increasing in Korea. According to the recent research, there are approximately between 1,040 to 1,380 commercial divers and between 1,180 to 1,900 diving fishermen and 300,000 more recreational divers in Korea [1-3]. The number of divers is increasing at estimated rate of 20,000 per year in Korea [3]. Divers are exposed to a lot of risk factors as a result of the underwater environment. Injuries from these hazards include barotrauma, decompression sickness (DCS), toxic effects of hyperbaric gases, drowning, hypothermia, and dangerous marine animals. Failure to accurately diagnose and appropriately treat some diving injuries can result in fatal outcome [4].

There are few underwater and hyperbaric medicine (UHM) specialists in Korea [3]. From occupational and environmental medicine (OEM) specialist’s perspective, we believe that UHM should be a branch of the OEM because OEM specialist can comprehend diving medicine and access the field of UHM more easily than specialist of different major. The reasons for this are the followings.

First, it is required for diving medicine physician (DMP) to know pressure related diseases and aquatic diseases such as hypothermia, marine animal injuries in the underwater environment. OEM specialist can readily understand these because injuries caused by physical and biological hazards are specific academic field of OEM.

Secondly, DMP must know about the toxicity of diving gases including nitrogen, oxygen, carbon dioxide and carbon monoxide. It is not difficult for OEM specialist to know about them because they have learned clinical toxicology.

Thirdly, many divers are undertaken for scientific, seafood harvesting, construction, maintenance, filming, military, forensic, and rescue purposes. In diving activities associated with occupational purposes, OEM specialist can have a better understanding of UHM. If DMP are to exactly evaluate the individual diver in relation to the requirements and hazards of a particular working environment, they must have a good knowledge of the tasks and the risks of the job.

Fourthly, the main medical practices of OEM specialist are special health examination and assessments of fitness to work. Divers need special health examination to ensure that they maintain an adequate level of fitness.

For these reasons, we think that OEM specialist is cut out for DMP.

Since May 2011, we’ve experienced of 122 cases as decompression illness (DCI) at Boryeong public health center and treated with hyperbaric oxygen chamber. This article will review and update on UHM including barotrauma, DCI, toxicity of diving gases and fitness for diving, thus help to extend our knowledge about UHM.

## Review

### The physical and physiologycal effects of the underwater environment

The most significant change in the underwater environment is the increased ambient pressure. Pressure increases in linear fashion with depth (Table 1). For each 10 meters (m), the underwater pressure increases by one atmospheric pressure [5]. Since most diving related injuries are a consequence of the behavior of gases under change of pressure, it is important to remember the two relevant gas laws of physics.

**Table 1:** Pressure change and lung volume with depth

**Depth**	**Depth**	**Absolute pressure**	**Lung volume**
**(fsw)**	**(m)**	**(ata)**	**(%)**
0	0	1	100
33	10	2	50
66	20	3	33
99	30	4	25
132	40	5	20
165	50	6	16.7

fsw (feet sea water), ata (atmospheres absolute pressure).

The first is Boyle’s law related to compression and expansion of gases. It states that at constant temperature, the volume is inversely proportional to the absolute pressure (P_1_V_1_ = P_2_V_2_). For example, an average adult male diver’s lungs may contain about 6 liters (ℓ) of gas [6]. If a breath-hold diver takes a full breath at the surface and descends to 10 m, the volume of air in his lungs may be reduced from 6 ℓ to 3 ℓ. Inversely, if a diver takes a full breath at 10 m from his scuba set and returns to the surface without exhaling, the volume of gas in his lungs will increase from 6 ℓ to 12 ℓ [7].

The second is Henry’s law, it states that the quantity of gas which will dissolve in a liquid at a given temperature is proportional to the partial pressure of gas in contact with the liquid. The greater the partial pressure of the gas, the greater the amount of gas that will dissolve into solution. Whenever a diver descends, more nitrogen will dissolve in the body because the partial pressure of nitrogen in the air being breathed is increased. During decompression, the decrease in ambient pressure may exceed the elimination rate of nitrogen, resulting in tissue supersaturation and formation of bubbles [8]. Bubbles can form in any tissue in the body including blood and have mechanical, embolic, and biochemical effects.

### Barotruma

Barotrauma is defined as tissue damage resulting from the mechanical effects of pressure. During ascent and descent, divers are exposed to changes in ambient pressure. For a diver to prevent barotrauma, the pressure in air-filled organs must be equalized to ambient pressure. Otherwise, underwater pressure forces blood and tissue fluids into the air-filled organs during descent until ambient pressure is realized. Similarly, expansion of trapped air causes barotrauma during ascent [9].

### Ear barotrauma

**External ear**: If external ear canal is obstructed, the enclosed air can result in barotrauma to the canal and tympanic membrane (TM) during descent and ascent. With such blockage, ear canal pressure becomes negative during descent or positive during ascent. Obstruction of the canal can be caused by cerumen, foreign bodies, the use of earplugs, the use of tight fitting hood, and bony growths (exostoses). The resulting tissue damages include congestion, hemorrhage, outward or inward bulging, and possible rupture of TM.

**Middle ear**: The most common diving medical problem is middle ear barotrauma, occurring in 30% of first-time divers and 10% of experienced divers [4]. It is resulting from inadequate pressure equilibration between the middle ear and the external environment. It occurs more commonly during descent and results from failure to actively open the normally closed eustachian tube [9]. If the diver fails to equalize, hydrostatic pressure will force the TM inwards, stretching it and increasing pain. At the same time, reduced air volume in the middle ear is compensated by blood and tissue fluid, causing edema of the middle ear mucosa. Ultimately the blood vessels become over distended and rupture, bleeding into the TM and the middle ear space [10]. At the other extreme, the TM may tear or rupture. With TM rupture, pain usually is severe and vertigo can occur from caloric effect if water enters the middle ear. Treatment of middle ear barotrauma is generally symptomatic. Prevention is best accomplished through the use of a Valsalva maneuver to let air into the middle ears via the Eustachian tubes [4].

**Inner ear**: Although inner ear barotrauma occurs infrequently, it may lead to acute sensorineural hearing loss, tinnitus and dizziness. Forceful attempts to equalize the middle ear with the Valsalva maneuver may elicit a pressure gradient between the middle ear and the inner ear. If the pressure gradient is large enough it may rupture the round or oval window, causing perilymph leakage. The treatment is bed rest, head elevation and referral to an otolaryngologist [4].

### Sinus barotrauma

Sinus barotrauma is another relatively common diving injury usually resulting from sinus outflow compromise, such as inflammatory mucosal thickening, polyps, and structural deviations. The pressure in the obstructed sinus remains relatively low during descent, resulting in a vacuum effect. This effect may be stressful to the sinus mucosal lining and may cause mucosal edema, serosanguineous exudate, and submucosal hematoma [11]. These may cause facial pain, epistaxis, and numbness due to pressure on branches of the trigeminal nerve in the maxillary sinus [9]. The diagnosis of sinus barotrauma is usually made clinically, and imaging is not necessary. Most of the patient required no treatment, but some cases can be helped by using topical and systemic decongestants, and appropriate antibiotics.

### Pulmonary barotrauma

Pulmonary barotrauma is one of the most serious diving accidents. It results from overexpansion of the lungs when divers can’t properly ventilate the expanding pulmonary gas. In the most severe cases, air will enter the bloodstream via pulmonary veins, traveling through the left heart as an arterial gas embolism (AGE). Differential alveolar compliance results in differential expansion of adjacent lung units causing tearing between vessels and airways and rupture of small airways or alveoli. Escaping gas can enter the pleural cavity (pneumothorax), mediastinum (mediastinal emphysema), pericardial cavity (pneumopericardium), or pulmonary veins (which transits the left heart to result in arterial gas embolism) [12]. Treatment is symptomatic, or 100% oxygen may be administered by mask to promote more rapid resolution of signs and symptoms. Pneumothorax, depending on its size, is treated with 100% oxygen or a chest tube.

### Decompression illness

DCI is a term that covers both DCS and AGE. It was introduced because treatment of the two conditions is recompression. Distinguishing AGE from DCS may be unnecessary for the clinical management of DCI, but such distinction remain a valid goal for understanding cause, pathology and prognosis and for improving therapy and decompression procedures. Diagnostic certainty is not required because divers with suspected DCI should be recompressed if there are no medical contraindications and a chamber is available [13].

### Arterial gas embolism

AGE is the most dangerous diving disorder, and can occur by rapid ascent, breath-holding, or the presence of lung disease. It happens in two distinct ways, one is due to pulmonary barotrauma, the other is due to cardiac DCS [14]. AGE due to pulmonary barotrauma arises when expanding gas stretches and ruptures alveolar capillaries allowing alveolar gas to enter the blood circulation [15]. This syndrome can be caused by gas becoming trapped as a result of airways obstruction in disorders such as asthma [16] or by the presence of pulmonary blebs, cysts, or bullae [17]. AGE due to cardiac DCS is discussed in part of DCS.

AGE most often affects the brain but can occasionally affect the heart and other organs. Arterial air embolism usually involves intracranial vessels and results in a catastrophic neurologic emergency. Clinical features include stroke-like symptoms (unconsciousness, motor and sensory changes, seizures) during ascent or within a few minutes after surfacing from a compressed gas dive. Other organ systems, such as the heart, can also be affected, but the clinical diagnosis of AGE is not reliable without CNS manifestations [13]. Most cases improve partially or completely over a short period as blood flow is re-established, but relapse is common over the hours following due to re-embolisation [12]. Of note, the greatest change in lung volume per change in depth occurs nearest the surface. Therefore, it is possible for divers breathing compressed air in a pool as shallow as 4 feet to develop AGE if they ascend to the surface while holding their breath at maximum lung volume [18].

### Decompression sickness

DCS is the syndromes associated with the formation and increase in size of extravascular and intravascular bubbles when the partial pressure of inert gas in the blood and tissues exceeds ambient pressure [13]. Gas bubbles cause mechanical tissue compression or embolisation to venous blood vessels by an expanding bubble volume, creating tissue ischemia, and edema [13]. Although the occurrence of DCS is not easily predictable, many predisposing factors are known. Major risk factors include dive depth, duration, rate of ascent, and repetitive dive. Other risk factors involve low ambient temperature, exposure to altitude, patent foramen ovale, female gender, old age, obesity, alcohols consumption, dehydration, previous DCS, and strenuous exercise.

While some instances of DCS (especially in saturation diving) occur during decompression, most cases present soon after surfacing. Although AGE can arise after ascent from very shallow depths, DCS almost never occurs after a single dive to depths of less than 6 m, even for an extended time [19], and is uncommon at depths of less than 10 m.

DCS has a wide range of manifestations, reflecting the effects of bubble formation in various anatomical locations [12]. It may be trivial, but can be fatal resulting in permanent paralysis and death. In the past it was traditional to describe DCS as type I (mild) and Type II (more serious or complicated). Recently DCS is classified with the clinical features according to the organ or system involved [20].

**Musculoskeletal DCS:** The most common symptom of DCS called “bends” is joint pain, however other types of pain which do not involve joints may occur. The examination of a patient with pain only DCS usually reveals no evidence of joint inflammation. The shoulder is most often affected while the elbows, wrists, hands, hips, knees and ankles are less frequent [20]. The pain may be reduced by bending the joint to find a more comfortable position. It may be located in a joint or muscle, may increase in intensity, and is usually described as a deep and dull ache. If not treated, pain usually continues for several uncomfortable days before slowly subsiding [13]. In mild cases, minor discomfort lasting only a few hours (“niggles”) may be the only manifestation.

**Neurological DCS:** DCS can affect the brain, spinal cord or peripheral nerves. The bubbles may be located in or near the blood vessels supplying the brain, causing obstruction of blood flow and direct pressure on the neurological tissues. The onset of cerebral DCS is often headache due to brain swelling. Numbness, tingling, weakness, paralysis, difficulty with speech, visual disturbances, confusion, amnesia, unconsciousness, or seizures are all possible presenting symptoms of this disorder. Spinal DCS is probably the most dangerous form of the commonly encountered syndromes of DCS [21]. Disturbances in movement such as weakness or paralysis or disturbances in sensation such as numbness or tingling are common. Interference with nerve supply to the bladder or intestines can cause urinary or fecal incontinence.

**Pulmonary DCS:** Small quantities of venous gas emboli are common in diving although they are usually asymptomatic because they are effectively filtered by the lung vessels. However, profuse venous gas emboli can overcome the pulmonary capillary filter, leads to obstruct the blood flows, can cause cough, dyspnea, and pulmonary edema. Frequently the symptoms occur very soon after ascent, from relatively deep dives (over 30 m) or after prolonged dives. Increasing of lung congestion may progress to complete circulatory collapse, loss of consciousness, and death if recompression is not administered immediately.

**Cardiac DCS:** Patent foramen ovale or other right-to-left cardiac shunt is present in about 30% of the normal population, and theoretically some venous gas emboli could enter the arterial circulation and reach the CNS, where they could grow from the inward diffusion of supersaturated inert gas [22]. When large amounts of gas emboli obstruct the lungs, the back pressure in the right atrium can exceed the pressure in the left atrium. Then patent foramen ovale may open allowing gas bubbles to pass from the right to the left side of the heart. As a result, gas emboli being pumped can lead to AGE with a different mechanism compared to pulmonary barotrauma. Bubbles can occasionally pass down the coronary arteries, restricting the blood supply to the heart itself. In severe instances this can lead to myocardial infarction.

**Gastrointestinal DCS:** Obstruction of blood flow to the intestines by gas bubbles can occasionally affect the gut. Clinical features are not common, but can include vomiting or diarrhea, abdominal distension or pain and hemorrhage into the gut. Severe cases can show clinical shock, and can bleed to death.

**Audiovestibular DCS:** The symptoms of audiovestibular DCS include loss of balance, tinnitus, hearing loss, vertigo, dizziness, nausea, and vomiting. Audiovestibular DCS should be differentiated from inner ear barotrauma, since the treatments are different. “Staggers” has been used as another name for audiovestibular DCS because of the afflicted diver’s difficulty in walking due to vestibular system dysfunction. However, symptoms of imbalance may also be due to neurological DCS involving the cerebellum. Typically, rapid involuntary eye movement (nystagmus) is present in audiovestibular DCS, but not in cerebellar DCS.

**Cutaneous DCS:** The most common skin manifestation of DCS is itching and skin rashes. This condition is not serious and requires no treatment. In more severe DCS, bubbles in the blood can obstruct blood supply to the skin, causing mottling or marbling of the skin, known as cutis marmorata.

**Lymphatic DCS:** Lymphatic obstruction may occur, creating localized pain in involved lymph nodes and swelling of the tissues drained by these nodes. Recompression may provide prompt relief from pain. The swelling, however, may take longer to resolve completely and may still be present at the completion of treatment [23].

**Constitutional DCS:** Headache, unexplained fatigue, malaise, and poorly localized aches are common observations in many cases of DCS. The precise cause of the constitutional DCS is unknown, but this condition is unlikely to be explained by bubble formation in any discrete location. The most plausible explanation for constitutional symptoms arises from the inflammatory effects of bubbles in whatever location they form [24].

### Diagnosis of DCI

Although the nonspecific nature of DCI makes diagnosis difficult, rapid diagnosis offer these patients the best chance of survival with minimal complications. Diagnosis depends on clinical basis, thus accurate history and physical examination including neurological examination is essential for initial assessment of DCI. Detailed history including data on decompression procedure, absolute pressure attained and breathing gas composition are all helpful for differential diagnosis [25].

Some laboratory abnormalities have been described in DCI, but there are no specific blood markers of the disease. Serum creatine phosphokinase can be elevated in AGE (predominantly the MM and MB isoenzymes), presumably because of myocardial or skeletal muscle injury. Severe DCI can be accompanied by hemoconcentration due to damage of the capillary endothelium with capillary leak, and fluid loss from intravascular space [26]. Thus, complete blood cell count including hemoglobin or packed-cell volume could help guide fluid resuscitation. Abnormalities on chest radiographs include pulmonary edema in cardiorespiratory DCS [27], focal opacities due to aspiration of water or vomitus, and pulmonary overdistension. Bubbles are rarely detectable with radiography in joints affected by pain, and are seldom observed in the brain or spine with either MRI or CT [28,29]. Imaging studies are not recommended for initial assessment because they postpone the time to treatment, except for conditions that require different therapy, such as hemorrhage or pneumothorax [15,25]. Audiometry and electronystagmography for audiovestibular DCS can usually be postponed until after recompression. Doppler ultrasonography and echocardiography are useful for research into venous gas emboli [30], but not for diagnosis of DCI. Several disorders that DCI may be confused are listed in Table 2. The differential diagnosis of DCI includes acute neurological disorders such as stroke and seizure.

**Table 2:** Differential diagnosis of decompression illness

**System**	**Conditions that can mimic decompression illness**
Otorhinolaryngology	Inner-ear barotrauma
	Middle ear or sinus barotrauma with cranial nerve compression
Neurology	Acute coincidental neurological disorder (stroke, seizure, subarachnoid hemorrhage)
	Guillain-Barre syndrome
	Migraine
	Multiple sclerosis
Hematology	Porphyria
Pulmonalogy	Immersion pulmonary edema
	Pneumothorax
Psychology	Acute psychosis
Other	Contaminated diving gas and oxygen toxic effects
	Musculoskeletal Strains or trauma sustained before, during, or after diving
	Near drowning and hypoxic brain injury
	Seafood toxin poisoning
	Ingestion
	Ciguatera
	Puffer fish
	Paralytic shellfish

### Treatment of DCI

Depending on the amount and location of excess intracorporeal gases, symptoms of DCI may resolve spontaneously in some cases [31]. Many patients will later deteriorate, often to a worse condition than their initial symptoms, as a result of progressive tissue damage. Thus, patients affected by DCI must receive adequate treatment promptly [25]. The principles of basic and advanced life support apply to any emergency of DCI. In addition to normal first aid procedures, 100% oxygen (O_2_) administration is a priority. Oxygen not only treats arterial hypoxemia but also enhances the rate of removal of inert gas and the elimination of bubbles. Although the head-down position is no longer routinely recommended because it can promote cerebral edema [32], the supine position has advantages over upright posture because it may prevent postural hypotension and enhance inert gas washout [33]. Depending on the patient’s level of consciousness, mild DCI may be managed by oral rehydration; otherwise, intravenous administration of 1–2 ℓ of fluid during the first hour post-injury is recommended, followed by a constant infusion of 1.5 mℓ/kg per h [34]. Glucose-containing fluids are avoided because the hyperglycemia can worsen CNS injury [35], and hypotonic fluids should not be used because they promote intracellular edema [36].

**Figure 1:**
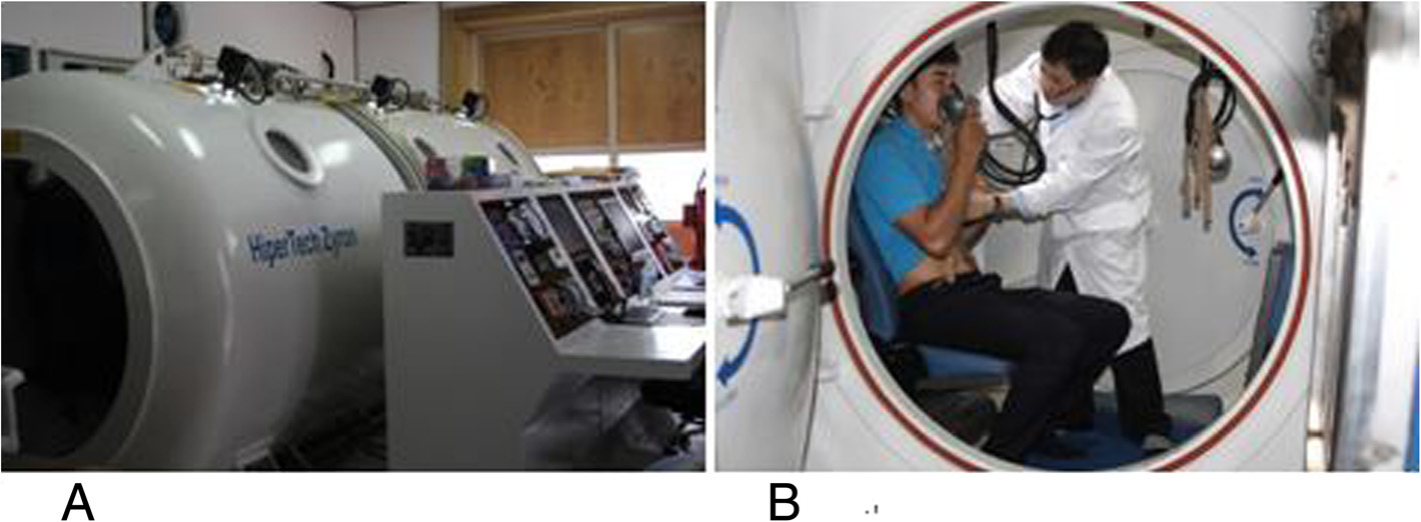
**Recompression chamber. (A)** Multiplace recompression chamber in Boryeong public health center, **(B)** Emergency treatment in a multiplace chamber.

**Figure 2:**
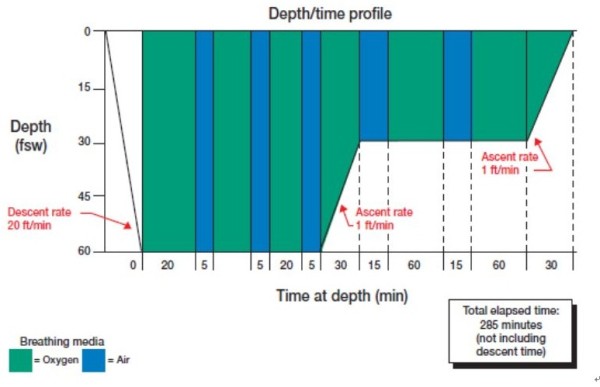
**US navy treatment 6.** Three pure oxygen breathing cycles of 20 min duration each at 60 fsw are followed by subsequent oxygen cycles at 30 fsw. Intermittent 5-min air breathing pauses prevent oxygen toxicity. Total treatment time is 285 min.

The definitive and effective treatment is recompression therapy. Compression physically reduces bubble volume in accordance with Boyle’s law, thus, alleviate DCI symptoms. The use of 100% oxygen as the breathing gas during recompression is therapeutic via various mechanisms including rapid elimination of inert gas, maximal oxygenation of ischemic tissues, reduction of edema, and inhibition of secondary inflammatory and reperfusion injury [37]. Recompression (Figure 1) should accomplish as soon as possible to avoid late recurrence and increased severity unless another cause is obvious. Recompression therapy is usually advised even if manifestations resolve with first treatment since untreated DCI can recur days after the initial onset [38]. Recompression schedules for the treatment of DCI consist of a rapid recompression to a specified pressure with oxygen breathing interrupted periodically by “air breaks” and continuing during a subsequent slow and staged decompression. The most common Recompression schedule is US Navy Treatment 6 (Figure 2) that comprises cycles of oxygen breathing at 60 fsw and 30 fsw with a total recompression time of approximately 4 h 45 min. If symptoms do not resolve completely, Recompression is repeated once or twice daily, until the symptoms are relieved or a plateau in improvement is reached.

Specific drugs have been used as adjuncts to recompression. Non-steroidal anti-inflammatory drugs can reduce the number of recompression sessions required, although symptomatic outcome was not improved [39].

### Toxicity of diving gases

Although a gas supply is essential for survival in the underwater environment, breathing gas under conditions of increased partial pressures may cause complications. There is no ideal gas mixture and the choice of diving gases is determined by balancing the physiological effects of each gas and the diving types. The commonly used gas mixtures include compressed air, 100% oxygen, nitrox (nitrogen–oxygen mixture), heliox (helium–oxygen mixture) and trimix (helium-oxygen-nitrogen mixture). Below 50 m, heliox or trimix usually replace air as a breathing gas, both to avoid nitrogen narcosis and to reduce density [12]. The clinical syndromes associated with diving gases are discussed below.

### Nitrogen narcosis

When breathed under atmospheric pressure, nitrogen has nontoxic effect, whereas it has narcotic effects when breathed under increased pressure. Nitrogen narcosis is characterised by temporo-spatial disorientation, memory troubles, euphoria, hallucinations, mood changes, impaired neuromuscular coordination and impaired intellectual performance. Sign and symptoms are first noticed at about 30 m (4 ata) and become increasingly more severe as depth increase. The relation of depth to narcosis is sometimes informally known as “Martini’s law”, the idea that narcosis results in the feeling of one martini for every 10 m below 20 m depth.

The management of narcosis is simply to ascend to shallower depths, then the effects disappear within minutes. Also the most straightforward way to avoid nitrogen narcosis is for a diver to limit the depth of dives. Narcosis while deep diving can be prevented by breathing with a gas mixture containing helium.

### High pressure nervous syndrome

High pressure nervous syndrome (HPNS) is a neurological and physiological diving disorder that results when a diver descends below 120 m while breathing a heliox. Symptoms of HPNS include tremor, myoclonic jerks, somnolence, EEG change, and decreased mental performance due to membrane and neurotransmitter mediated effects of pressure [40]. The severity of HPNS can be alleviated by several methods. These include choice of a suitable slow exponential rate of compression, use of long stages or holds during compression to allow adaptation, use of trimix and selection of the least susceptible divers [41].

### Oxygen toxicity

It is paradoxical that oxygen is essential to sustain life to divers, but toxic under hyperbaric condition. Although exact causes of oxygen toxicity are not yet known, it is generally considered that free radical intermediates (superoxide anions, hydrogen peroxide, hydroperoxy and hydroxyl radicals, and singlet oxygen) play an important role in oxygen toxicity. Free radical intermediates are potentially toxic to cell membranes, enzymes, nucleic acids, and other cellular constituents [42]. The major factors affecting oxygen toxicity are the concentration of the gas used, the duration of the exposure, and the susceptibility of the individual person. The two common forms of oxygen toxicity affect the lungs and the brain.

**Pulmonary toxicity:** The early symptoms of pulmonary oxygen toxicity are substernal irritation, progressing to pain and a burning sensation in parallel with increasing coughing. In severe cases, acute respiratory distress syndrome ensues and atelectasis supervenes [43]. The onset of symptoms varies but usually occurs after 12 to 16 h of exposure at 1.0 ata, 8 to 14 h at 1.5 ata, and 3 to 6 h at 2.0 ata. At oxygen pressures of 2.5 and 3.0 ata [44], pulmonary symptoms have earlier onset but are less severe because exposure durations are limited by neurologic manifestation of oxygen toxicity [45]. This toxicity will usually resolve spontaneously if the supplementary oxygen administration is ceased as soon as symptoms develop. If it is essential to continue oxygen therapy however, a reduction in the partial pressure of oxygen given will slow the development of toxicity. Long term hyperoxic exposure must generally be limited to around 0.4–0.5 ata in order to avoid pulmonary oxygen toxicity.

**Central nervous system toxicity:** Short term oxygen exposure can be much higher but exposure to partial pressures greater than 1.6 ata may cause acute central nervous system (CNS) toxicity. The manifestations of this include visual change, tinnitus, nausea, twitching (especially of the face), irritability, dizziness, and convulsions. Convulsions in divers are extremely hazardous, can lead to death by drowning.

### Breathing gas contamination

The supply of uncontaminated breathing gas is necessary for life to the diver because of the magnifying effect on contamination by the partial pressure rise with increasing depth. Contamination usually arises either from impurities in the air taken into the compressor or from contaminants generated by the compressor itself. Carbon monoxide, nitrogen oxides, oil, and hydrocarbon contamination is currently receiving attention as a possible cause of some cases of underwater incapacitation [46].

### Long term effects of diving

Long term health effects of diving involve neurocognitive dysfunction, dysbaric osteonecrosis, hearing loss and pulmonary function changes.

### Dysbaric osteonecrosis

Dysbaric osteonecrosis (DON) is known as a chronic complication of prolonged and repetitive diving, even though the exact mechanisms leading to bone death are still debated [47]. DON lesions usually affect the long bones with fatty marrow, most commonly humerus, femur and tibia. Lesions are classified into two groups according to the sites involved and radiological appearances. Those adjacent to the joint surface of the hips and shoulders are classified as “juxta-articular” or “type A” lesions. Lesions which are found in the head, neck, and shaft are classified as “type B” lesions [48]. Type A lesions are potentially crippling due to the eventual collapse of the joint surface, while type B lesions rarely cause symptoms. Direct radiography is the main screening and diagnostic method. No treatment is indicated for shaft lesions because they are not expected to produce symptoms or to result in disability [49]. The treatment of aseptic necrosis of bone at juxta-articular sites is not yet satisfactory. Severe cases may require the fusion of a joint or its replacement.

### Fitness for diving

Whether diving is considered a recreation or an occupation, performance and safety require a reasonable level of physical conditioning. In most countries, recreational diving is either minimally regulated or not regulated. Working divers must be medically fit to perform their varied tasks in safety, yet it is not a simple matter to define the criteria of fitness and to pinpoint the threshold of any abnormality that should be disqualifying. In the United States, Occupational Safety and Health Administration standards provide a list of contraindication to hyperbaric exposures [50] (Table 3). These include history of seizure disorder, chronic inability to equalize sinus or middle ear pressure, pneumothorax, cardiac abnormalities such as coronary artery disease and osteonecrosis. Detailed fitness to dive considerations and a more comprehensive list of conditions can be found in Guidelines for The European Diving Technology Committee webpage [51]. The examining doctor must not apply merely written standards or guidelines for medical assessment of divers. If a diver has some medical problem, the examining doctor must interpret the relevant standards in the relation to the requirements and hazards of the diver’s working environment and the need for underwater safety. Resumption of diving after a decompression injury needs medical assessment by DMP. The European Diving Technology Committee recommends minimum periods before considering a return to diving after decompression illness [51] (Table 4).

**Table 3:** List of contraindication to hyperbaric exposures in OSHA standards

**System**	**Disorders that may restrict or limit occupational exposure**
Neurology	History of seizure disorder other than early febrile convulsions
	Chronic inability to equalize sinus and/or middle ear pressure
Otorhinolaryngology	Meniere’s disease
	Vestibular end-organ destruction
Hematology	Hemoglobinopathies
Pulmonalogy	Cystic or cavitary disease of the lungs
	Obstructive or restrictive lung disease
	Pneumothorax
Cardiology	Cardiac abnormalities (e.g., pathologic heart block, valvular disease, intraventricular conduction defects other than isolated right bundle branch block, angina pectoris, arrhythmia, coronary artery disease)
Other	Malignancies (active) unless treated and without recurrence for 5 yr
	Impaired organ function caused by alcohol or drug use
	Conditions requiring continous medication for control
	(e.g., antihistamines, steroids, barbiturates, mood altering drugs, or insulin)

**Table 4:** Recommended times away from diving

**DCI type**		**Minimum time**
Simple DCI (limb pain, skin “bend”, lymphatic swelling, headache, fatigue etc.)	Uncomplicated recovery	24 hours
	Recurrence/relapse	7 days
	Altered sensation in limbs only	7 days
Neurological DCI	Other (audiovestibular, motor, pulmonary etc.)	28 days

## Conclusion

With the recent increase in underwater activity, UHM is a field of medicine with high growth potential. In addition, recompression therapy can be used in a wide variety of medical conditions, including osteoradionecrosis, skin grafts, gas gangrene, refractory osteomyelitis, radiation induced injury, poorly healing wounds, and acute traumatic ischemic injury [52]. Even though UHM is a practical and promising field of medicine, there are few institutes to train or research UHM in Korea. Consequently, most Korean physicians have scanty knowledge about UHM, as well as the lack of recompression facilities hinders DCI patients from getting treatment quickly. To solve these problems; physicians, diving related organization, and government should be concerned and further develop UHM. We hope that UHM will form a part of the field of OEM in Korea and this article will enlighten and challenge OEM specialist about UHM.

## Consent

Written informed consent was obtained from the patient for the publication of this report and any accompanying images.

## Abbreviations

OEM: Occupational and environmental medicine; DCS: Decompression sickness; UHM: Underwater and hyperbaric medicine; DMP: Diving medicine physician; DCI: Decompression illness; TM: Tympanic membrane; AGE: Arterial gas embolism; DON: Dysbaric osteonecrosis.

## Competing interests

The authors declare that they have no competing interests.

## Authors’ contributions

YIL and BJY conducted literature searching together. YIL drafted the initial manuscript, BJY corrected, edited and finalized it. Both authors read and approved the final manuscript.
